# Generation of cleidocranial dysplasia-specific human induced pluripotent stem cells in completely serum-, feeder-, and integration-free culture

**DOI:** 10.1007/s11626-015-9968-x

**Published:** 2015-11-11

**Authors:** Sachiko Yamasaki, Atsuko Hamada, Eri Akagi, Hirotaka Nakatao, Manami Ohtaka, Ken Nishimura, Mahito Nakanishi, Shigeaki Toratani, Tetsuji Okamoto

**Affiliations:** Department of Molecular Oral Medicine and Maxillofacial Surgery, Applied Life Sciences, Graduate Institute of Biomedical & Health Sciences, Hiroshima University, Kasumi 1-2-3, Minami-ku, Hiroshima, 734-8553 Japan; Research Center for Stem Cell Engineering, National Institute of Advanced Industrial Science and Technology (AIST), 1-1-1 Higashi, Central 4, Tsukuba, Ibaraki 305-8562 Japan; Laboratory of Gene Regulation, Graduate School of Comprehensive Human Sciences, University of Tsukuba, 1-1-1 Tennodai, Tsukuba-shi, Ibaraki 305-8575 Japan

**Keywords:** iPS cells, Serum-free, Feeder-free, Sendai Virus, Cleidocranial dysplasia

## Abstract

Human pluripotent stem cells hold great promise for their practical and scientific potentials. To improve understanding of self-renewal and differentiation, we previously reported a defined serum-free medium hESF9 could generate and maintain human induced pluripotent stem cells (iPSCs) in serum- and feeder-free culture conditions using retroviral vectors. To avoid the unpredictable side effects associated with retrovirus integration, we report here the successful generation of hiPSCs from dental pulp cells with a non-integrating replication-defective and persistent Sendai virus (SeVdp) vector expressing four key reprogramming genes. We found that hESF9 medium in combination with fibronectin are effective for generating and maintaining hiPSCs with SeVdp (KOSM). Using this system, pluripotent and self-renewing hiPSCs could be easily and stably generated and propagated. With this system, we successfully generated hiPSCs from cleidocranial dysplasia (CCD) caused by a heterozygous germ-line mutation of runt-related protein2 (RUNX2), which has an important role in the differentiation of osteoblasts and maturation of chondrocytes. This is the first report of the establishment of CCD-specific iPSCs. The cartilage in the teratomas of CCD-iPSCs showed abnormalities. These CCD-iPSCs would be beneficial to clarify the molecular mechanism and for development of medical applications. Moreover, it brings new pathophysiological role of RUNX2 in the differentiation of the human chondrocytes and osteocytes.

**Electronic supplementary material:**

The online version of this article (doi:10.1007/s11626-015-9968-x) contains supplementary material, which is available to authorized users.

## Introduction

The generation of induced pluripotent stem cells (iPSCs) by expression of exogenous defined factors provides valuable tools for transplantation therapies and regenerative medicine using patient-specific stem cells and also for understanding the mechanisms of human diseases. Human iPSCs were established originally by the introduction of the transcription factors *Oct4*, *Sox2*, *Klf4*, and *c-Myc* using a retroviral vector (Park et al. 2007; Takahashi et al. 2007). DNA-integrative retroviral and lentiviral vectors have been used widely because of the stable expression of transgenes owing to chromosomal insertion of the vector genome (Takahashi et al. 2007; Yu et al. 2007; Lowry et al. 2008). However, iPS technology is complicated by the potential risks posed by continuous expression of transgenes and by genome integration of viral vectors. Silencing the expression of exogenous transgenes is indispensable for maintaining pluripotency (Zhou and Zeng 2013), and genome-integrating viral vectors can produce insertional mutations, which may influence differentiation potential while reactivation of the c-Myc oncogene may lead to tumorigenesis (Okita et al. 2007). For these reasons, more efficient and safer reprogramming methods have been explored to generate iPSCs carrying no exogenous genetic material.

Recently, a number of procedures have been used to generate genetically non-integrative or unmodified human iPSCs. These approaches involve chemicals or plasmid, episomal or viral vectors (Kaji et al. 2009; Woltjen et al. 2009; Zhou et al. 2009; Jia et al. 2010; Warren et al. 2010; Zhu et al. 2010; Yu et al. 2011;). However, these approaches suffer from an extremely low efficiency in generating iPSCs, and require either chemical treatment or extended periods of transduction (Kim et al. 2009; Zhou et al. 2009; Bernal 2013).

Sendai virus (SeV) is a member of the Paramyxovirdae family, and is an enveloped virus with a single-stranded, negative-sense, and non-segmented RNA genome (Yoshida et al. 1979; Lamb and Kolakofsky 2001; Nishimura et al. 2007). As SeV does not infect humans but is pathogenic for various animal cells with an exceptionally broad host range, various applications have been studied with SeV as a recombinant viral vector capable of transient but strong gene expression (Nakanishi and Otsu 2012). Previously, we described a replication-defective and persistent Sendai virus (SeVdp) vector in a novel gene transfer/expression system (Nishimura et al. 2011; Nakanishi and Otsu 2012). The SeVdp vector can express up to four exogenous genes simultaneously at a fixed ratio, and it can be erased quickly by interfering vector-encoded RNA polymerase. These characteristics are ideal for generating high-quality, exogenous gene-free iPSCs, and we demonstrated that SeVdp installed with Yamanaka’s four factors could reprogram human tissue cells (Nishimura et al. 2011; Nakanishi and Otsu 2012).

Previously, we found that a hESF-GRO basal medium supplemented with a minimal growth factor defined serum-free culture medium hESF9 (Yamasaki et al. 2014), could generate and maintain hiPSCs. In this culture system, the effect of exogenous factors could be precisely analyzed without the confounding influences of unknown or undefined components. Therefore, with use of serum-free defined hESF9 medium and a fibronectin substrate alkaline phosphate (ALP)-positive hiPSCs were generated without feeder cells with high induction efficiencies. However, manipulation of iPSCs remained difficult because the self-renewing property of these cells was unstable. We found TGF-β1 promotes the growth of undifferentiated hiPSCs in hESF9 (Furue et al. 2008; Ohnuma et al. 2014; Yamasaki et al. 2014). However, conventional protocols for inducing hiPSCs (Park et al. 2007; Takahashi et al. 2007; Yu et al. 2007) required repetitive induction, or produced insufficient excision of integrated reprogramming vectors. Therefore, we sought an improved method to induce hiPSCs without retaining expression of reprogramming factors or integrated vector DNA for both basic studies and clinical applications. Here, we show that by using SeVdp, we efficiently generated viral/factor-free hiPSCs from dental pulp cells (DPCs) in serum- and feeder-free culture conditions.

We used this new method to reprogram adult human somatic cells from cleidocranial dysplasia (CCD) into iPSCs without feeder cells. CCD (MIM #119600) is a dominantly inherited disorder caused by mutation in the gene encoding transcription factor *RUNX2* (*Cbfa1*), which was mapped to chromosome 6p21. RUNX2 has an important role in the differentiation of osteoblasts and in the maturation of chondrocytes (Komori et al. 1997; Smith et al. 2005). The main clinical features of CCD include persistently open skull sutures with bulging calvaria, hypoplasia or aplasia of the clavicles permitting abnormal facility in opposing the shoulders, wide pubic symphysis, short middle phalanx of the fifth fingers, dental anomalies, and often vertebral malformation. RUNX2 has a primary role in the differentiation of osteoblasts and hypertrophy of cartilage at the growth plate, cell migration, and vascular invasion of bone (Cohen 2009). To our knowledge, this is the first report of the establishment of CCD-specific iPS cells. Here, we report molecular and phenotypic profiles of CCD in tissues differentiated from hiPSCs derived from patient’s dental pulp cells that carry a heritable mutation in *RUNX2*. The teratoma-containing cartilage from CCD-iPSCs revealed abnormality by histological analysis. Patient-derived iPSCs could provide somatic cells, which cannot be directly obtained from patients, and this advantage may contribute to understanding mechanisms of the disease and lead to the development of a new field of disease modeling or useful tools for exploring disease mechanisms.

## Materials and Methods

### Ethics statement and cell culture of dental pulp cell.

This study was approved by the Ethics Committee of Human Genome/Gene Analysis Research at Hiroshima University (approval number: hi-58). We obtained human third molars from healthy volunteers or supernumerary tooth from CCD patient (17-year-old girl) at Hiroshima University Hospital after obtaining informed consent for the use of dental pulp cells (DPCs) to generate iPSCs in accordance with the approved guidelines. The dental pulp tissues obtained aseptically from extracted human third molars or supernumerary tooth were minced into small clumps and cultured on type I collagen-coated dishes in RD6F serum-free medium (Yamasaki et al. 2014). Primary human DPCs were isolated and cultured at 37°C in a humid atmosphere of 95% air/5% CO_2_. For subculturing, DPCs were harvested in 0.05% trypsin-0.02% ethylenediamine tetraacetic acid (EDTA) in Ca^2+^- and Mg^2+^-free phosphate-buffered saline (CMF-PBS), and the trypsin was inactivated with 0.1% soybean trypsin inhibitor (Sigma Aldrich, St. Louis, MO).

### Induction of hiPSCs with SeVdp under completely defined culture.

Human DPCs cultured in a 12-well plate at a density of 1 × 10^5^ cells in RD6F serum-free medium were infected with SeVdp (KOSM) vector at MOIs of 3, 6, and 9, at room temperature for 2 h then at 37°C for overnight in a humid atmosphere of 95% air/5% CO_2_ in RD6F medium. Then, these infected cells were trypsinized and seeded on fibronectin (2 μg/cm^2^) (Sigma Aldrich)-coated dishes at 1.0×, 1.2×, 1.5×, 1.8×, and 2.0 × 10^4^ cells per six-well plate in hESF9-medium (Furue et al. 2008; Ohnuma et al. 2014; Yamasaki et al. 2014) at 38°C in a humid atmosphere of 95% air/5% CO_2_. The medium was changed every other day. Within 20 d after transduction, iPS colonies were picked based on human ES cell-like colony morphology. The picked colonies were mechanically dissociated into small clumps and subsequently expanded and maintained on fibronectin-coated dishes in hESF9 with TGF-β1 (2 ng/ml) (hESF9T) or activin A (10 ng/ml) (hESF9a) at 37°C, 95% air/5% CO_2_ as described previously (Hayashi et al. 2011; Yamasaki et al. 2014). We defined this stage as passage 1. The medium was changed every day with hESF9T or hESF9a medium. Reprogramming efficiency was determined as the positive number of total ALP positive colonies per total number of infected cells. At the same time, transduced DPCs were seeded on mitomycin-C-treated mouse embryonic fibroblast (MEF) cells with KSR-based conditions (Takahashi et al. 2007; Yu et al. 2007) as a control.

### Immunocytochemistry and alkaline phosphate (ALP) staining.

Cells were fixed with PBS containing 4% paraformaldehyde for 10 min at room temperature and washed with PBS. Continuously, cells were treated with PBS containing 5% normal goat or rabbit serum (Nichirei Biosciences Inc., Tokyo, Japan) and 0.1% Triton X-100 at room temperature. Then cells were fixed and stained with antibodies to Oct4 (1/200 Millipore, Billerica, MA), Tra-1-60 (1/200, Stemgent^®^, Cambridge, MA), Tra-1-81 (1/200, Stemgent^®^), SSEA-1 (1/100, Stemgent^®^), SSEA-4 (1/100, Stemgent^®^), MAP2 (1/200, Millipore), Nestin (1/200, Millipore), α-smooth muscle actin (α-SMA: pre-diluted, DAKO Cytomation, Glostrup, Denmark), α-fetoprotein (1/100, R&D Systems Minneapolis, MN), and to the SeV nucleocapsid protein (mouse monoclonal antibody, clone #2E4, 1.6 mg/mL). These primary antibodies were visualized with Alexa Fluor^®^ 488-conjugated goat anti-rabbit IgG, or Alexa Fluor^®^ 488-conjugated rabbit anti-mouse IgG, or Alexa Fluor^®^ 488-conjugated goat anti-mouse IgG (1/200, Invitrogen, Carlsbad, CA). Cell nuclei were stained with 4', 6-Diamidine-2'-phenylindole dihydrochloride (DAPI). Fluorescence images were taken using a Zeiss inverted LSM 700 confocal microscope (Carl Zeiss, GmbH, Germany). Alkaline phosphate (ALP) staining was performed using a Fast Red substrate kit (Nichirei) according to manufacturer’s instruction.

### RNA isolation and reverse transcription gene expression.

Total RNA was extracted from iPSCs using the Illustra RNA spin Mini Isolation kit (GE Healthcare UK Ltd, Buckinghamshire, England) according to manufacturer’s instruction. cDNA was synthesized from 1 μg of total RNA using High capacity RNA-to cDNA master mix (Applied Biosystems, Carlsbad, CA) and RT-PCR reactions were performed with KOD-FX neo (Toyobo, Japan) using primers described in Supplementary Table S2. PCR products were size-fractionated using 1.5% agarose gel electrophoresis. DNA markers were used to confirm the size of the fragments.

### Verification of removal of SeVdp-iPS genome.

Human iPSCs generated with SeVdp (SeV-iPSCs) and cultured in serum- and feeder-free culture conditions were harvested with a trypsin-EDTA, then total RNA was extracted. We carried out RT-PCR for detecting SeVdp NP mRNA.

### PCR analysis of genomic DNA and mutation analysis.

Genomic DNA was used for analysis of viral integration. iPSCs using QIAamp^®^ DNA mini kit (Qiagen, Valencia, CA) according to the manufacturer’s instructions. Genomic DNA was used for PCR reactions to check for viral integration using KOD-FX neo (Toyobo). Specific primer sets were used that detect only the transgene and not the endogenous gene. Reactions were performed with pMXs primers described in Supplementary Table S2.

Mutational analysis of *RUNX2* gene was performed by using specific primers franking the eight coding exons and their splice junctions (Quack et al. 1999). PCR products purified using PCR purification kit (QIAGEN) and sequenced directly using CEQ8000 sequencer (Beckman coulter, Brea, CA).

### In vitro differentiation of integration-free iPSCs.

Human iPSCs generated and maintained in serum- and feeder-free culture conditions with SeVdp (SeV-iPSCs or CCD-iPSCs) were differentiated in vitro by the formation of embryoid bodies (EBs) as described previously (Yamasaki et al. 2014). Briefly, undifferentiated hiPSCs were cultured in Dulbecco's Modified Eagle Medium (DMEM) supplemented with 10% fetal bovine serum for 4 d in low-attachment 96 well plates. After 4 d in suspension culture, aggregated EBs were harvested onto gelatin-coated dishes for another 10 d. Then, the EBs were fixed and stained with hematoxylin/eosin (H-E) staining and Alcian Blue/PAS staining. Histological findings were evaluated using a Nikon ECLIPSE E800 microscope (Nikon Corporation, Tokyo, Japan) and photographed using Leica DC500 (Leica Microsystems AG, Wetzlar, Germany).

### Assay for teratoma formation.

SeV-iPSCs maintained in serum- and feeder-free culture conditions were injected subcutaneously into dorsal flank of SCID (CB17/Icr-*Prkdc*^*scid*^/CrlCrlj) mice (1 × 10^6^ cells/50 μl of the cell suspension). Around 10 weeks after the injection, tumors were surgically dissected, weighed, fixed in PBS containing 4% paraformaldehyde, and embedded in paraffin. Each section was stained with hematoxylin/eosin staining, Alcian Blue/PAS staining, and toluidine blue staining. Histological findings were evaluated as described above. All animal experiments in this study were strictly followed a protocol approved by the Institutional Animal Care and Use Committee of Hiroshima University (approval number: A-11-140).

### Chondrogenic differentiation.

For in vitro chondrogenic differentiation, CCD-iPSCs were transferred to low-attachment 96-well plates with hMSC Chondrogenic Differentiation medium (Lonza, Basel, Switzerland) supplemented with dexamethasone, ascorbate, ITS supplement, GA-1000, sodium pyruvate, proline, l-glutamine, and TGF-β3 (R&D Systems), at a cell density of 1 × 10^5^ cells/well in 200 μl. After 3 d in suspension culture, embryoid bodies were transferred to 24-well low-attachment plates. Medium was changed every 3 d. After 10 weeks, particles were fixed in 4% (*w*/*v*) paraformaldehyde and embedded in paraffin and the content of sulfated glycosaminoglycans (GAGs) was investigated by Alcian blue/PAS staining (Quarto et al. 2012; Yamashita et al. 2014; Okada et al. 2015). The data are representative of three independent experiments.

### Short tandem repeat DNA analysis.

Genomic DNA was used for PCR with Powerplex 16 system (Promega Corporation, Madison, WI) and analyzed by ABI PRISM 3100 Genetic analyzer and Gene Mapper v3.5 (Applied Biosystems).

### Karyotyping.

Standard Q-banding chromosome analysis was carried out using quinacrine and Hoechst 33258 as described previously (Murakami et al. 2013; Lin et al. 1978). Approximately 50 separate metaphase spreads of SeV-iPSCs maintained in serum- and feeder-free culture conditions were examined using a Zeiss Axio Imager microscope (Carl Zeiss) and mapped.

## Results

### Generation of integration-free hiPSCs in serum- and feeder-free culture.

**Figure 1.**
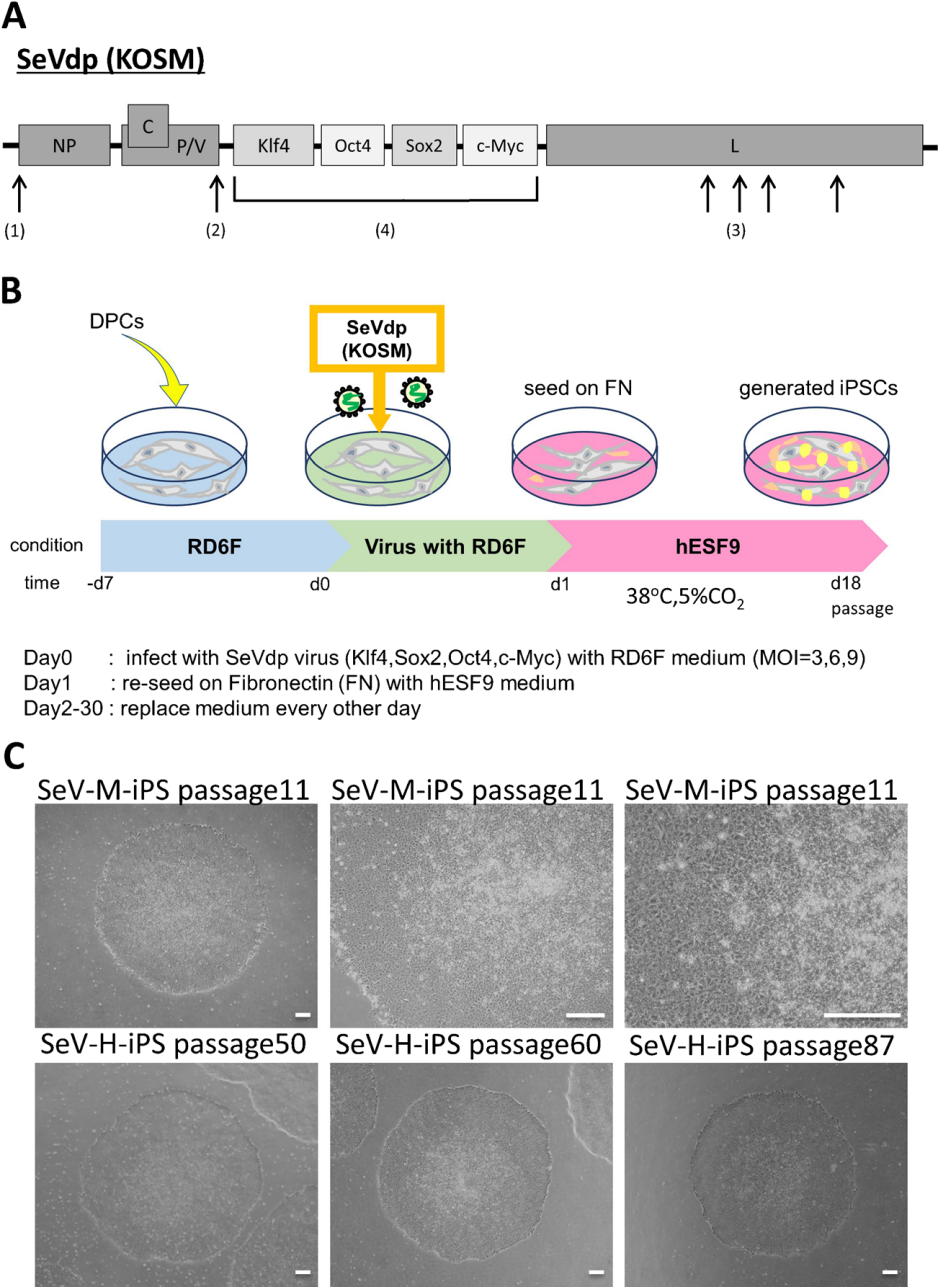
Integration-free hiPS cell generation from DPCs in serum- and feeder-free culture conditions using SeVdp. (*A*) Genome structure of defective persistent Sendai virus (SeVdp) vector. SeVdp has mutations in the L and P genes, which are responsible for low cytotoxicity and for defective induction of IFN-b. The M, F and HN genes are deleted and replaced with genes interest (KOSM). SeVdp-iPS was installed with Klf4, Oct4, Sox2, c-Myc cDNA on a single vector. (*1*) Insertion of Gene-End signal, (*2*) Mutation in P gene (P517H), (*3*) Mutation in L gene (V981I, S1088A, C1207S, V1618L) (*4*) Deletion of M, F and HN genes, and installation of exogenous genes. (*B*) Time schedule of hiPS cell generation. *Day −7∼0* DPCs were cultured in RD6F serum-free medium on type I collagen coated dish. *Day 0∼1* SeVdp (KOSM) transduction (*Oct4, Sox2, Klf-4, c-Myc*) with hESF9 medium. *Day 1* re-seeding on fibronectin-coated plate with hESF9 medium. *Day 2∼30* exchange medium every other day. (*C*) Phase contrast images of iPSCs derived from normal DPCs (SeV-M-iPS and SeV-H-iPS). (*upper panels*) SeV-M-iPS clone1 at passage 11 on fibronectin-coated dish with hESF9 medium. (*lower panels*) SeV-H-iPS-clone25 at passage 50, clone35 at passage 60 and passage 87 on fibronectin-coated dish with hESF9 medium. *Bars* indicate 200 μm. DPC-H-derived iPS cells generated with Sendai virus were designated SeV-H-iPS and DPC-M-derived iPS cells were designated SeV-M-iPS.

To avoid transgene integration, we used a SeVdp (KOSM) vector, which does not integrate into the host genome and is not pathogenic for humans. Moreover, providing a significant advantage for cell reprogramming, transcription factors (*Klf4*, *Oct4*, *Sox2*, and *c-Myc*) were incorporated into a single vector allowing these genes to be expressed reproducibly at fixed ratios (Fig. 1*A*). We carefully dissected dental pulp tissues from third molar teeth and cultured dental pulp cells (DPCs) in a serum-free defined medium designated RD6F (Sato et al. 1987; Myoken et al. 1989) (Fig. 1*B*, Supplementary Fig. S1A). DPCs less than two passages were used for hiPS cell reprogramming with the SeVdp (KOSM) vector at MOIs of 3, 6, and 9 in completely feeder- and serum-free culture conditions (Supplementary Fig. S2B). The infected DPCs were seeded on fibronectin-coated plate at 1.0 × 10^4^ cells per six-well plate in hESF9 medium at 38°C in a humid atmosphere of 95% air/5% CO_2_. After 7 d, ES-like tightly packed colonies appeared. Continuously, human iPS cell colonies appeared at 15 d after transfection. After 20 d in culture, individual iPS cell colonies were picked and subsequently passaged and maintained in hESF9T or hESF9a medium (Yamasaki et al. 2014; Ohnuma et al. 2014; Hayashi et al. 2011) in dishes coated with fibronectin at 37°C in a humid atmosphere of 5% CO_2_ (Fig. 1*C*). On 30 d in culture, alkaline phosphatase (ALP)-positive hiPS cell colonies were counted; approximately 24∼37 colonies were observed at MOI 3 (the efficiency of reprogramming was 0.30%) and 25∼42 colonies at MOI 6 (0.31%) and 15∼36 colonies at MOI 9 (0.25%) per six-well plate (Supplementary Fig. S2C).

### Generation of integration-free CCD-iPSCs.

**Figure 2.**
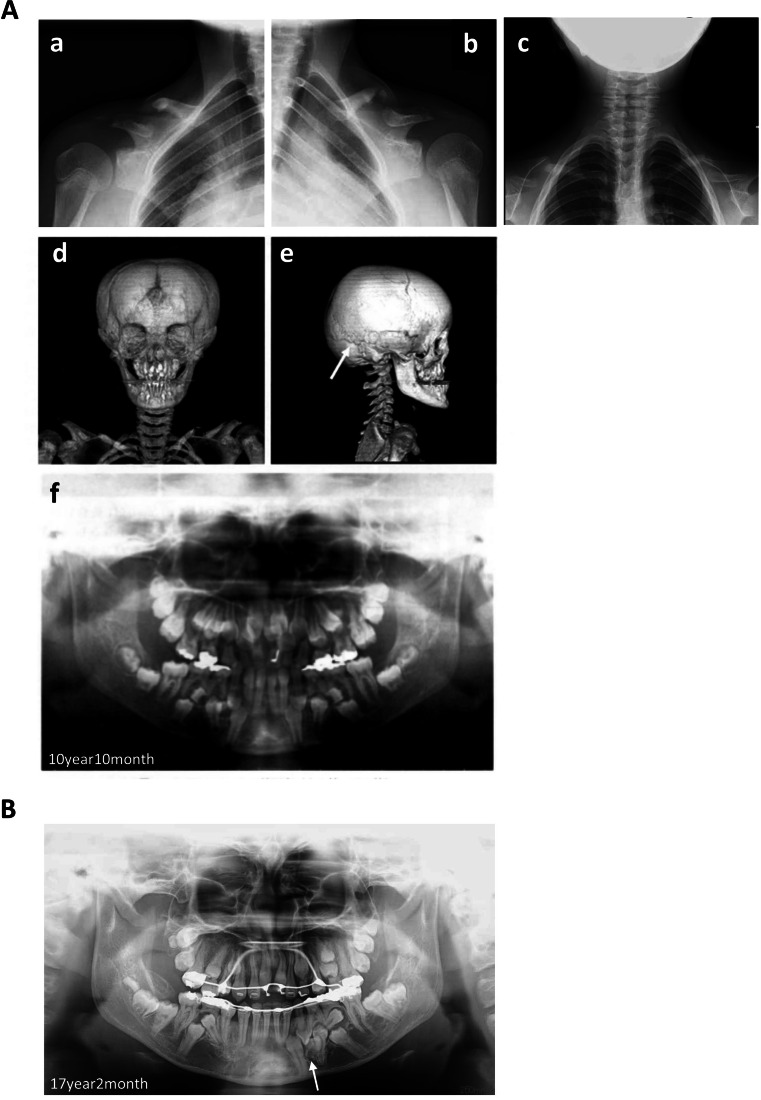
Typical morphologies from CCD donor (*A*) Typical radiological morphologies from CCD donor at the initial visit (*10 years10months*) (*a*) Chest radiograph showed partially absent clavicles. (*right side*) (*b*) Chest radiograph showed partially absent clavicles. (*left side*) (*c*) Chest radiograph revealed a cone-shaped chest, high position of the scapula, and bilateral clavicles were discontinuous. (*d*) Head computerized tomography showed open skull sutures, persistent open anterior fontanelle. (*e*) Head computerized tomography showed wormian bone and hypoplasia of the zygomatic arches. (*f*) Panoramic view showed delayed eruption of permanent teeth and supernumerary teeth at 10 yr and 10 mo (initial visit). (*B*) Panoramic view showed retention of deciduous teeth, delayed eruption of permanent teeth and many impacted supernumerary teeth at 17 yr and 2 mo. We obtained supernumerary tooth of mandible (*arrow head*).

**Figure 3.**
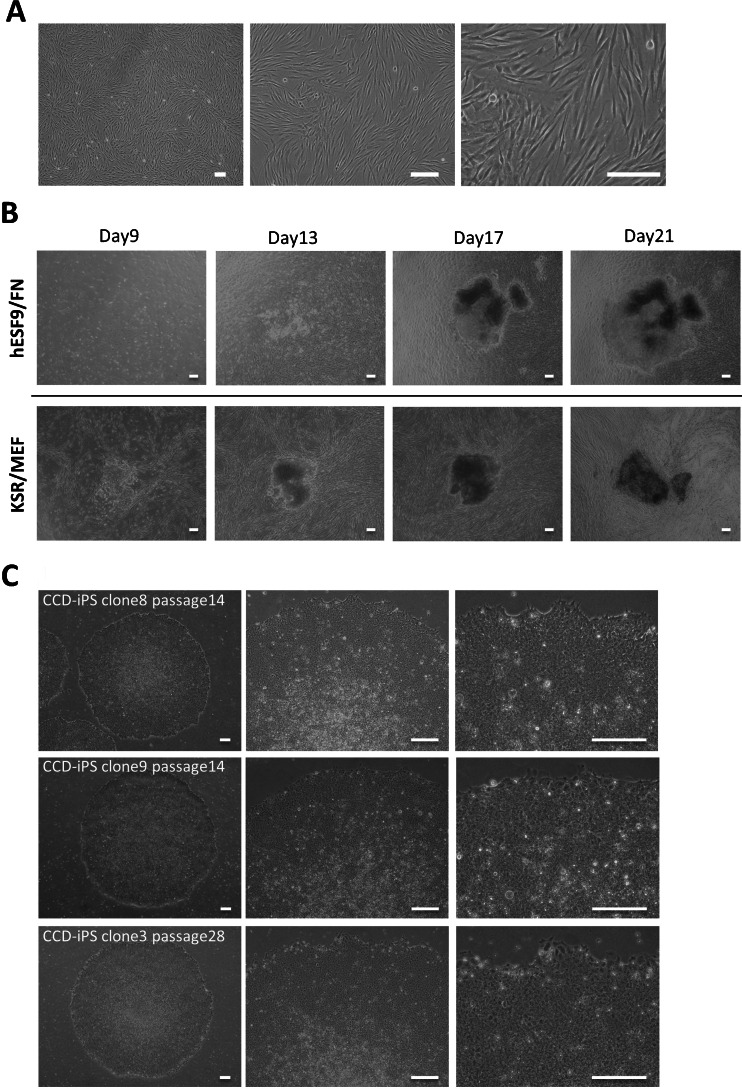
Generation of CCD-iPSCs in serum-, feeder-, and integration-free culture conditions. (*A*) Images of CCD-DPCs (passage 1) on type I collagen coated plate with RD6F medium. (*B*) Transduced CCD-DPCs were cultured on fibronectin (FN) with hESF9 medium or on MEF with KSR-based conditions at MOI = 6. After 22 d, colonies were picked up and sub-cultured on fibronectin. (*C*) Phase contrast images of iPSCs derived from CCD-DPCs (CCD-SeV-iPS1) clone8 at passage 14, clone9 at passage 14, and clone3 at passage 28. *Bars* indicate 200 μm.

A CCD patient was diagnosed with mutation of *RUNX2* (674G > A, R225Q), leading to a clinical phenotype (Fig. 2). We obtained CCD-DPCs from supernumerary tooth (Fig. 3*A*). CCD-DPCs were reprogrammed into iPSCs with SeVdp (KOSM) vector at MOI of 6 in completely serum-free culture conditions. The infected CCD-DPCs were seeded on fibronectin-coated plate at 1.0 × 10^4^ cells per six-well plate in hESF9 (Fig. 3*B*). After 22 d in culture, individual iPS cell colonies were picked and subsequently passaged and maintained in hESF9-based medium (Fig. 3*C*). The efficiency of reprogramming was 0.15% in hESF9.

### Characterization of integration-free hiPSCs derived from DPCs.

**Figure 4.**
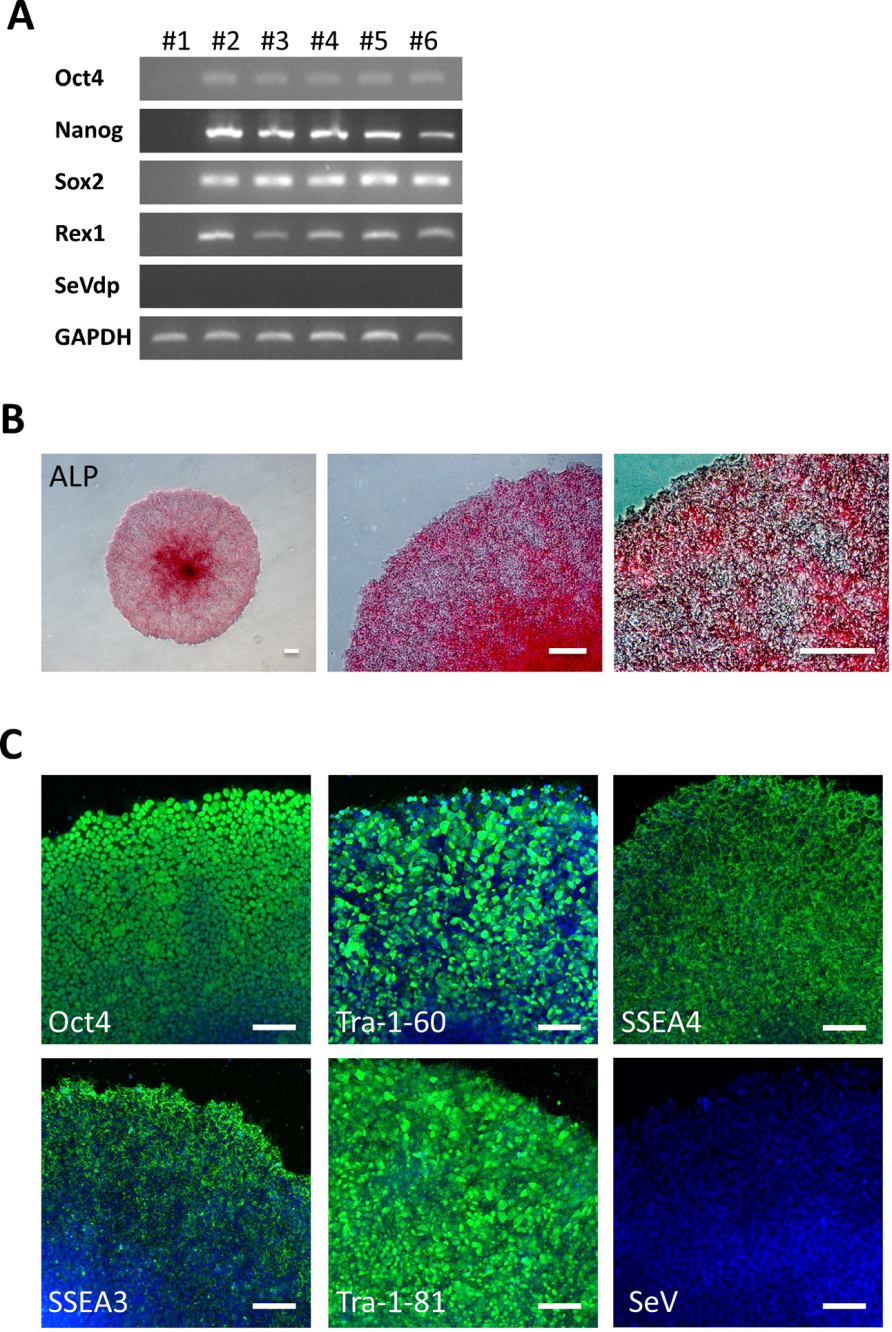
Characterization of CCD-iPSCs generated in serum-, feeder-, and integration-free culture conditions. (*A*) Expression of ES cell marker genes in iPSCs derived from CCD-DPCs. We used primers that only amplified the endogenous genes (Takahashi et al. 2007; Nishimura et al. 2007; Yamasaki et al. 2014). (*#1*) CCD-DPC: passage 1 = before infection (*#2*) CCD-SeV-iPS1 clone3: passage 11 = serum-free condition (*#3*) CCD-SeV-iPS1 clone4: passage 12 = serum-free condition (*#4*) CCD-SeV-iPS1 clone7: passage 13 = serum-free condition (*#5*) CCD-SeV-iPS1 clone8: passage 12 = serum-free condition (*#6*): CCD-SeV-iPS1 clone1: passage 7 = serum-supplemented condition CCD-DPC-derived iPS cells were designated CCD-SeV-iPS. Full-length blots are shown in Supplementary Figure S7. (*B*) ALP activity of generated CCD-iPSCs The ALP activity was detected (CCD-SeV-iPS1 clone3 at passage 68). *Bars* indicate 200 μm. (*C*) Immunocytochemistry of pluripotency marker proteins CCD-SeV-iPS1-clone3 grown under hESF9-based culture conditions for 42 passages were fixed and reacted with antibodies (Oct4, Tra-1-60 and Tra-1-81, SSEA-4, SeVdp). Binding of these antibodies was visualized with Alexa Fluor® 488-conjugated secondary antibodies (*green*). Nuclei were stained with DAPI (*blue*). *Scale bars* represent 100 μm.

**Figure 5.**
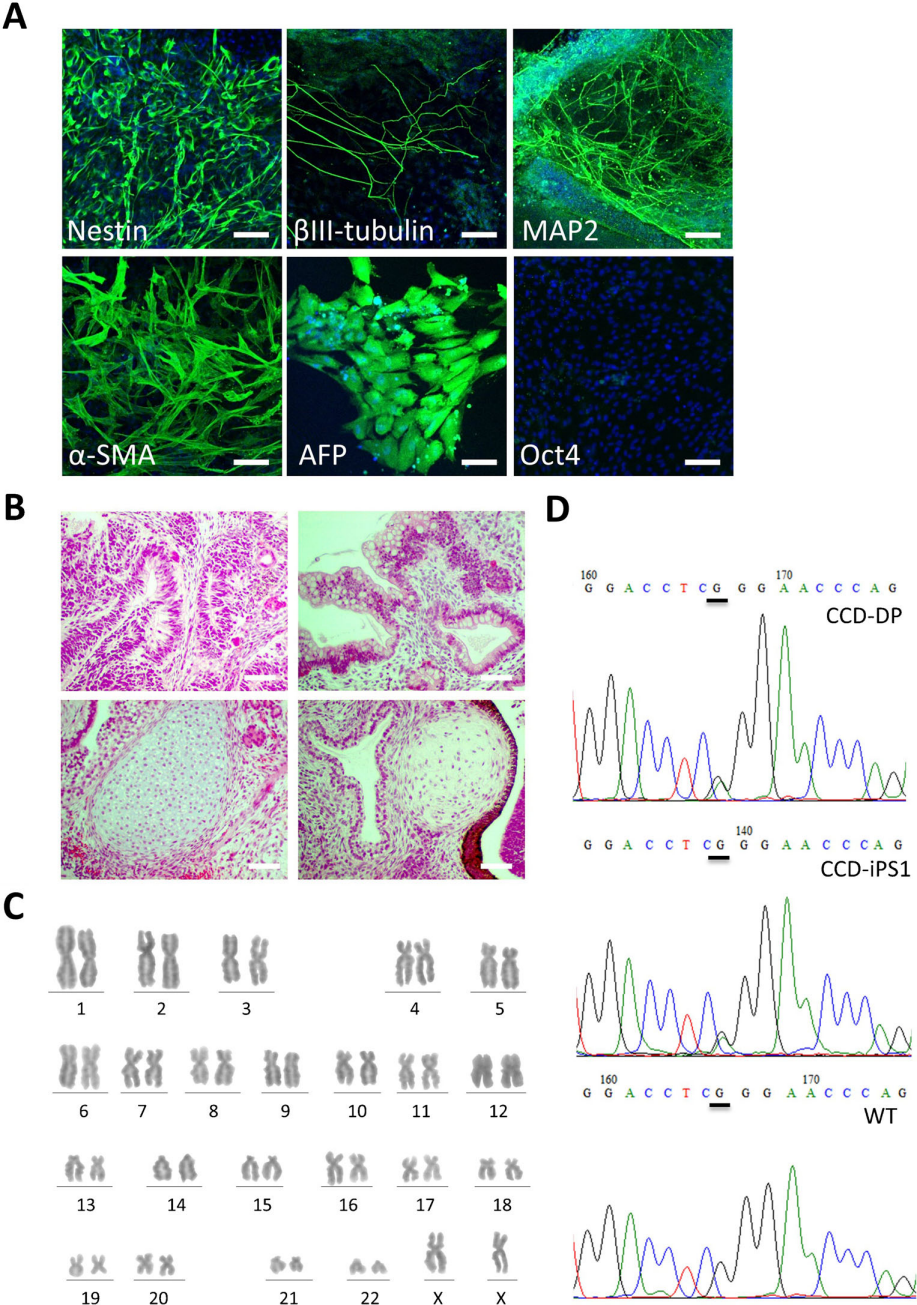
Differentiation ability of CCD-iPSCs derived from DPCs in serum-, feeder- and integration-free defined culture conditions. (*A*) Differentiation was performed using embryoid body formation, and the differentiated iPSCs (CCD-SeV-iPS1 clone8) were fixed and reacted with antibodies. Shown were immunocytochemistry of Nestin, βIII-tubulin, MAP-2, α-smooth muscle actin (*α-SMA*), and α-fetoprotein (*AFP*). Binding of these antibodies was visualized with Alexa Fluor® 488-conjugated secondary antibodies (*green*). Nucleuses were stained with DAPI. (passage 27) *Bar* indicates 100 μm. (*B*) Teratoma formation of CCD-iPSCs in the defined culture conditions in SCID mice. Teratomas were generated in SCID mice (CB17/Icr-*Prkdc* ^*scid*^/CrlCrlj) from CCD-SeV-iPS1 (clone3 at passage 28). Histological analysis with H-E staining demonstrated that teratomas formed from CCD-iPSCs cultured in hESF9-based conditions contained derivatives of all three germ layers. *Scale bars* represent 200 μm. (*C*) Karyotype analysis of CCD-SeV-iPS1 clone3 at passage 48 generated in hESF9 had a normal diploid 46, XX karyotype. (*D*) Sequencing result of dental pulp cell of CCD donor (*CCD-DPC*) and generated CCD-iPS cell (*CCD-iPS1*) demonstrated heterozygous mutation in *RUNX2*. The DPC carried a missense mutation (647G → A) which was responsible for arginine to glutamine substitution [R225Q]. *WT*: wild type (WT) allele pattern in *RUNX2.*

Both SeV-iPS and CCD-SeV-iPS cell clone generated as described above, continuously proliferated under serum- and feeder-free culture conditions for more than 100 passages. Reverse transcription-polymerase chain reaction (RT-PCR) analysis revealed that the iPSCs expressed endogenous pluripotent marker genes such as *Oct4*, *Sox2*, *Nanog*, *Esg1*, and *Rex-1*, whereas differentiated markers such as glial fibrillary acidic protein (GFAP) and α-fetoprotein (AFP) were not expressed (Fig. 4*A*, Supplementary Fig. S2A, S7). Using immunocytochemistry, these generated cells expressed Oct4, Tra-1-60, Tra-1-81, SSEA-4, and exhibited ALP activity, whereas they did not react with anti-SSEA-1 (Fig. 4*B*, *C*, Supplementary Fig. S2B, S2C). Using an in vitro differentiation assay involving embryoid body formation, we confirmed the differentiation potential of generated hiPSCs. After 2 weeks of induction, these embryoid bodies were immunoreactive with Nestin and MAP2 (ectoderm markers), α-smooth muscle actin (SMA) (mesoderm marker), and α-fetoprotein (AFP) (primitive endoderm marker), suggesting their differentiation potential to three germ layers (Fig. 5*A*, Supplementary Fig. S3A, S7). Human iPSCs generated and cultured in hESF9 on fibronectin formed teratomas consisting of three germ layers including neural tissues (ectoderm), epithelium (ectoderm), cartilage (mesoderm), and intestinal epithelial tissues (endoderm) in severe combined immunodeficient (SCID) mice (Fig. 5*B*, Supplementary Fig. S3B). Moreover, RT-PCR and immunohistochemical analysis failed to detect SeVdp (KOSM) genomic DNA or SeV nucleocapsid protein in the hiPSCs (Fig. 4*A*, *C*, Supplementary Fig. S2C, S4A).

### Short tandem repeat analysis and karyotype analysis.

Short tandem repeat (STR) analysis confirmed that the DPCs and generated iPSCs had the same STR allele pattern (Supplementary Table S1). Moreover, Q-band karyotype analysis of 50 randomly selected iPSCs at passage 92 and freshly generated hiPSCs possessed normal 46, XX karyotypes (Fig. 5*C*, Supplementary Fig. S4B). Moreover, selected CCD-iPS clone3 at passage 48 possessed normal 46, XX karyotypes (Fig. 5*C*).

### Missense mutations.

Missense mutations were detected in exon3 of *Runnx2* (Fig. 2*B*). The dental pulp cell and the CCD-iPSCs were both shown to carry a missense mutation (647G > A) in runt domain of *RUNX2*, which was responsible for arginine to glutamine substitution [R225Q] (Fig. 5*D*).

### Dysplasia of cartilage in CCD-iPSCs-induced teratomas of SCID mice.

**Figure 6.**
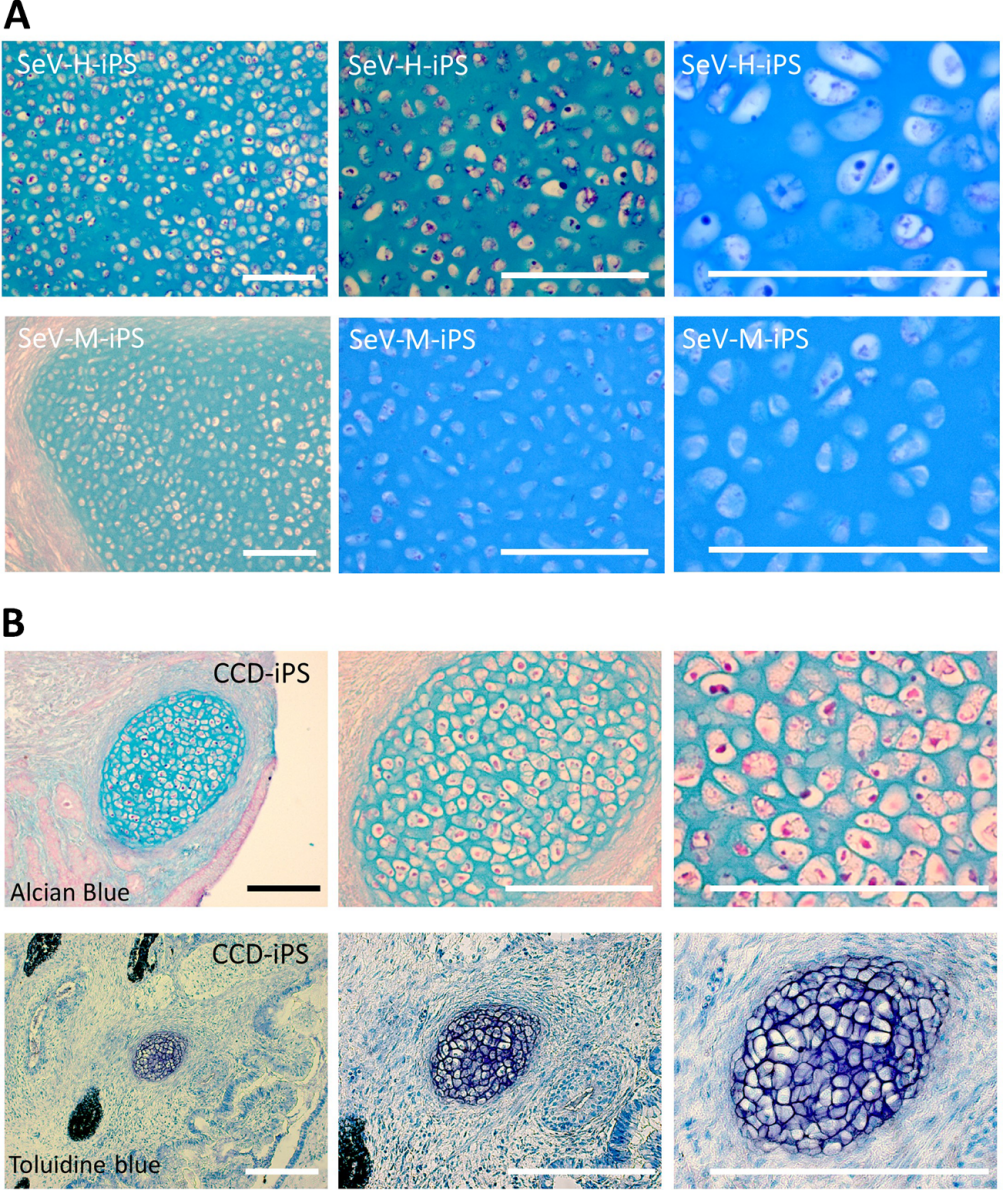
Examination of the cartilage formed in teratomas. (*A*) Alcian blue/PAS staining of the cartilage of teratomas generated by the injection of SeV-H-iPSCs (clone25 at passage 24) and SeV-M-iPSCs (clone6 at passage 14). (*B*) Alcian blue/PAS and toluidine blue staining of the cartilage of teratomas generated by the injection of CCD-SeV-iPSC1 (clone3 at passage 28). A histological analysis of the teratomas derived from CCD-iPSCs showed specific cartilage formation that the cartilage matrix tended to insufficient compare to the teratomas generated from control SeV-iPSCs (SeV-H-iPSCs and SeV-M-iPSCs) confirmed by stain with Alcian blue/PAS and toluidine blue staining. *Scale bars* represent 200 μm.

To understand pathogenesis of CCD-iPSCs, we evaluated their chondrogenic differentiation in teratomas of SCID mice. We examined the cartilage formed in teratomas. A histological analysis of the teratomas derived from CCD-iPSCs showed specific cartilage formation that the cartilage matrix tended to insufficiently compare to the teratomas generated from control SeV-iPSCs (SeV-H-iPS, SeV-M-iPS) confirmed by stain with Alcian blue/PAS and toluidine blue (Fig. 6).

### Impaired chondrogenic differentiation in CCD-iPSCs.

**Figure 7.**
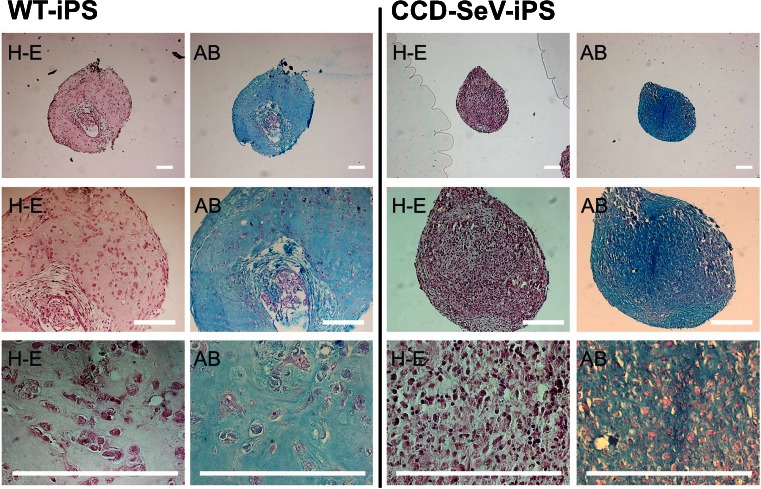
Examination of a histological analysis of particles formed chondrogenically differentiated WT-iPSCs or CCD-iPSCs. Wild type (WT)-iPSCs or CCD-iPSCs were chondrogenically differentiated for 10 wk. The images are representative of three independent experiments. After 10 weeks, CCD-iPSCs (*right side*) indicated significant difference by H-E and Alcian blue/PAS staining compared to wild type (*left side*). *Left side* WT-iPSCs (JCRB1331 passage 92) *Right side* CCD-iPSCs (CCD-SeV-iPS1clone3 passage 27). *Scale bars* represent 200 μm.

To identify the differentiation ability of CCD-iPSCs, we evaluated their chondrogenic differentiation. The results of the histological analysis of the pellet culture of chondrogenically differentiated CCD-iPSCs indicated striking difference in chondrogenesis confirmed by Alcian blue/PAS staining (Fig. 7). The particle of CCD-iPSCs indicated lack of cartilaginous elements.

## Discussion

As initially reported by Takahashi et al., hiPSCs were first established by introducing the transcription factors *Oct4, Sox2, Klf4*, and *c-Myc* into differentiated cells using a retroviral vector (Takahashi et al. 2007). Subsequently, researchers have made substantial efforts to establish methods for generating iPSCs by different approaches including gene transfer, protein transduction, and treatment with chemical compounds. Efficient cell reprogramming systems generally required the simultaneous and sustained overexpression of several reprogramming factors (Nishimura et al. 2007). However, after reprogramming had been completed, insertional vectors such as retroviral and lentiviral vectors were liabilities because integrated reprogramming transgenes could be reactivated, which might have affected the differentiation potential of iPSCs, and the random integration of the vectors into host cell genomes could potentially cause deleterious mutations. Both of these characteristics posed significant potential safety issues for future clinical uses of hiPS cells. The SeV vector described here expresses reprogramming genes without chromosomal integration. Defective and persistent Sendai virus (SeVdp) vectors are recognized as a superior tool for iPS cell generation because of their remarkably high potential and simplicity (Nishimura et al. 2011). As the four reprogramming genes are incorporated in a single vector, simultaneous delivery of four exogenous genes led to stable and reproducible expression of the genes at a pre-determined ratio without chromosomal integration. The rapid and complete loss of the vector genome after several passages makes feasible applications in the fields of gene therapy and the development of patient-specific therapeutic reagents.

In a previous study, we have reported generation of hiPSCs from adult human dental pulp cells (DPCs) and fibroblast using retrovirus and successfully maintained hiPSCs in an undifferentiated state for long-term period using serum-free defined medium without feeder cells (Yamasaki et al. 2014). Here, we described that integration-free hiPSCs could be generated from human DPCs using this complete serum-free culture system. Under this culture system, we successfully generated genome integration-free iPSCs from DPCs using SeVdp vector installed with *Klf4, Oct4, Sox2,* and *c-Myc*. These integration-free iPSCs could be generated from DPCs in 2∼3 weeks with SeVdp (KOSM) at a high efficiency, and exhibited similar morphology and cellular characteristics to those of established in conventional serum-supplemented culture conditions. These generated iPSCs could differentiate into several cell types such as neuronal cells, muscle cells, cartilage tissues, and intestinal epithelium.

We have derived human iPSCs from cleidocranial dysplasia (CCD) donors to understand the mechanisms underlying this disease. CCD is an autosomal dominant inheritance caused by heterozygous mutation in the Runt-related transcription factor 2 (*RUNX2*) gene on chromosome 6p21 (Zheng et al. 2005; Komori 2007). The main clinical features of CCD include persistently open skull sutures with bulging of the calvaria, hypoplasia, or aplasia of the clavicles permitting abnormal facility in opposing shoulders, short middle phalanx of the fifth fingers, abnormal dentition, and often vertebral malformation (Otto et al. 1997; Quack et al. 1999; Bufalino et al. 2011). RUNX2 is an important regulator of osteoblast differentiation, chondrocyte maturation, especially hypertrophy of cartilage at growth plate (Komori et al. 1997; Sugawara et al. 2011). This gene encodes a transcription factor, which has a DNA-binding Runt domain. Most CCD patients are reported to have mutations in this domain (Komori et al. 1997). In *RUNX2*-deficient mice, chondrocyte differentiation was severely disturbed with a lack of osteoblasts and bone formation (Komori et al. 1997; Yoshida et al. 2002). The mutations consist of missense, nonsense, frameshift, and splice mutations (Cohen 2009). These mutations of the *RUNX2* gene affect its downstream target (Otto et al. 2003). However, the effect of *RUNX2* mutations from CCD patients is still unclear.

In this study, donors showed features characteristic of CCD such as partially absent clavicles, persistent open anterior fontanelle, short stature, delayed eruption of permanent teeth, and supernumerary teeth (Banshodani et al. 2007). They also carried the missense mutation 674G > A in exon 3 of the *RUNX2*, which was predicted to substitute an arginine with a glutamine residue at codon 225 of the RUNX2 protein (Zheng et al. 2005). This mutation has been frequently identified and reported in several previous studies (Quack et al. 1999; Zhou et al. 1999; Otto et al. 2002; Kim et al. 2006; Xuan et al. 2008; Wu et al. 2014). Amino acid R225 is located in the c-terminal region of the Runt domain, which has been shown to be associated with severe parietal bone dysplasia (Cunningham et al. 2006). Substitution of R225 with glutamine is predicted to impair DNA binding to RUNX2. The Runt domain mediates binding to CBFβ, an unrelated partner protein that does not interact directly with DNA but enhances the DNA-binding affinity of the RUNX protein (Bartfeld et al. 2002). The mutations at this codon interfere with nuclear localization (NLS) and abolish DNA binding. Therefore this mutation (R225Q) may affect the DNA-binding activity of RUNX2 (Quack et al. 1999; Yoshida et al. 2002). Lack of nuclear RUNX2 accumulation might be the causes of haploinsufficiency in CCD.

In our studies, altered chondrocyte hypertrophy in teratomas formed by human CCD-iPSCs was found by histological analysis. Cartilage matrix revealed sparse and cytoplasm tended to be swelling and lack of normal hypertrophic chondrocytes with aggregation of small round cell has also been observed in the teratomas generated from CCD-iPSCs compared with healthy control. These findings suggest that this phenomenon may occur by inhibitory mechanisms of proliferation and differentiation of growth cartilage cells into hypertrophic chondrocytes mediated by *RUNX2* mutation. This histological analysis of teratomas suggests that the CCD-iPSCs model could mimic the pathology of CCD to some extent. Inhibition of chondrocyte differentiation and lack of osteoblasts and abnormal endochondral ossification might occur as a result of the disequilibrium of chondrocytes maturation in growth plate in CCD. Moreover, abnormal chondrocyte hypertrophy may associate with downregulation of *RUNX2* transcriptional targets.

CCD-iPSCs might be effective for screening small molecules that target different stages of human chondrogenesis, osteogenesis, tooth formation, and morphogenesis. They could help facilitate identification of the triggers of chondrogenic maturation and bone formation. In addition, CCD-iPSCs provide the possibility to create important human cell types that could not be directly obtained from CCD patients. CCD-iPSCs would also provide interesting human-specific perspectives complementary to the in vivo studies of the CCD mouse model. The mechanisms of tooth morphogenesis and formation are known to be strictly governed by cellular communications and the genetic control regulating signal pathways (Thesleff 2006; Xuan et al. 2010). Mutational and functional analyses in our CCD-iPSC model will help to identify the distinct effects of RUNX2 on chondrogenesis, osteogenesis, and tooth morphogenesis in the pathogenesis of CCD.

Our simple defined serum- and feeder-free culture system consisting of basal medium, defined components and growth factors along with a SeVdp vector can be helpful to understand specific signaling pathways and molecular mechanisms of cell reprogramming. In addition, our results suggest that generating patient-specific iPSCs with integration-free and serum-free methods could eliminate the possibility of complications resulting from persistent transgene expression. iPSC-derived cell types could be used to identify specific roles for RUNX2 in chondrocytes formation and to identify new therapeutic targets that would complement strategies to modulate the signaling properties of the RUNX2. Moreover, in this defined serum-free culture system, microRNAs and small RNAs produced by the iPSCs themselves could be easily purified for functional studies. In practice, we have generated a variety of disease-specific iPSCs from peripheral blood mononuclear cells using this culture method (data not shown).

## Conclusions

We have successfully generated transgene-free hiPSCs from human DPCs using SeVdp vector and maintained in an undifferentiated state in complete feeder- and serum-free defined culture. Using this culture system, we have derived disease-specific iPS cells from CCD patients. CCD-iPSCs are a valuable in vitro human model for understanding the mechanisms involving human chondrocytes in bone and tooth formation. Further characterization of the cells in the serum-, feeder-, and integration-free culture would be beneficial to clarify the molecular mechanism involved in the disease.

## Electronic supplementary material

ESM 1 (DOC 8320 kb)
