# Supplementary material for: Generation of cleidocranial dysplasia-specific human induced pluripotent stem cells in completely serum-, feeder-, and integration-free culture
Source: In Vitro Cell Dev Biol Anim. 2015 Nov 11;52:252–64. doi: 10.1007/s11626-015-9968-x (PMC4746228; doi:10.1007/s11626-015-9968-x)
Supplement: Supplementary file 1 — (DOC 8320 kb) [file 11626_2015_9968_MOESM1_ESM.doc]

# Supplementary Information

**Generation of Cleidocranial dysplasia-specific human induced pluripotent stem cells in completely serum-, feeder-, and integration-free culture**

Sachiko YAMASAKI ^a^, Atsuko HAMADA ^a^, Eri AKAGI ^a^, Hirotaka NAKATAO ^a^, Manami OHTAKA ^b^,

Ken NISHIMURA ^c^, Mahito NAKANISHI ^b^, Shigeaki TORATANI ^a^, Tetsuji OKAMOTO ^a *^

^a^ Department of Molecular Oral Medicine and Maxillofacial Surgery, Applied Life Sciences, Graduate

Institute of Biomedical & Health Sciences, Hiroshima University, Kasumi 1-2-3,

Minami-ku, Hiroshima 734-8553, Japan

^b^ Research Center for Stem Cell Engineering, National Institute of Advanced Industrial Science and

Technology (AIST), 1-1-1 Higashi, Central 4, Tsukuba, Ibaraki, 305-8562, Japan.

^C^ Laboratory of Gene Regulation, Graduate School of Comprehensive Human Sciences, University of

Tsukuba, 1-1-1 Tennodai, Tsukuba-shi, Ibaraki 305-8575, JAPAN

*Correspondence should be addressed to:

Tetsuji OKAMOTO, D.D.S., PhD. Department of Molecular Oral Medicine and Maxillofacial Surgery,

Applied Life Sciences, Institute of Biomedical & Health Sciences, Hiroshima University

Tel: +81-82-257-5665, Fax: +81-82-257-5669, e-mail: [tetsuok@hiroshima-u.ac.jp](mailto:tetsuok@hiroshima-u.ac.jp)

**Supplementary Figure1**


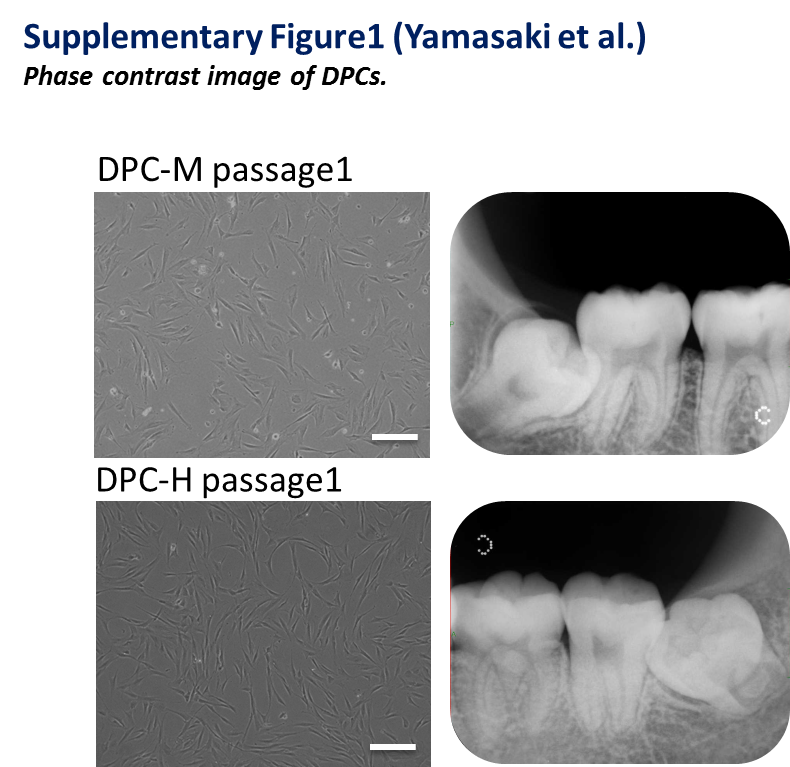
**Phase contrast image of DPCs.**

Figure S1: Phase contrast image of DPCs.

Images of DPCs (DPC-M and DPC-H: passage 1) on type I collagen coated plate with RD6F

medium and dental x-ray image of impacted third molar teeth.

Bars indicate 200 μm.

**Supplementary Figure2**

***hiPSC generation from DPCs in serum- and feeder-free culture conditions***


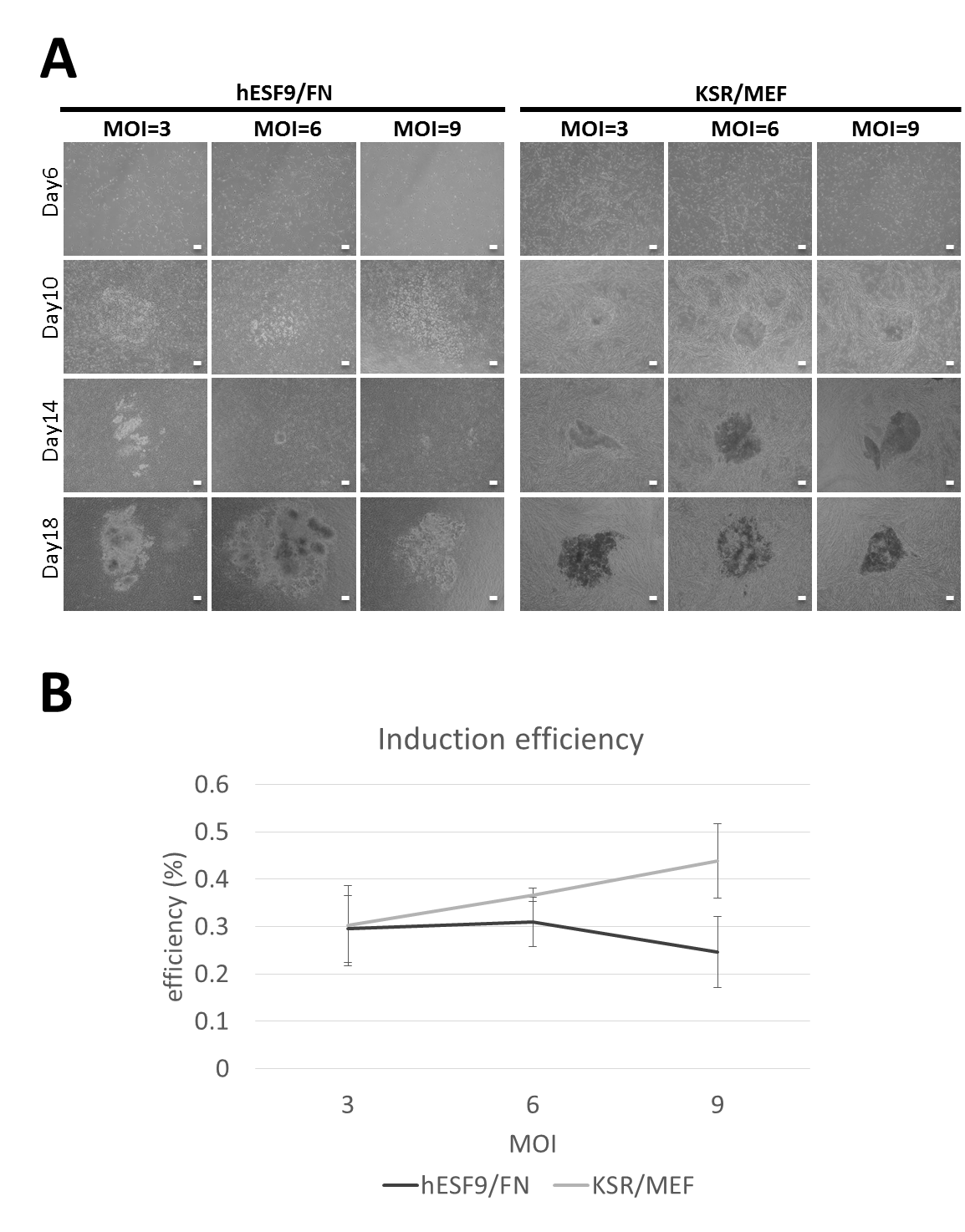


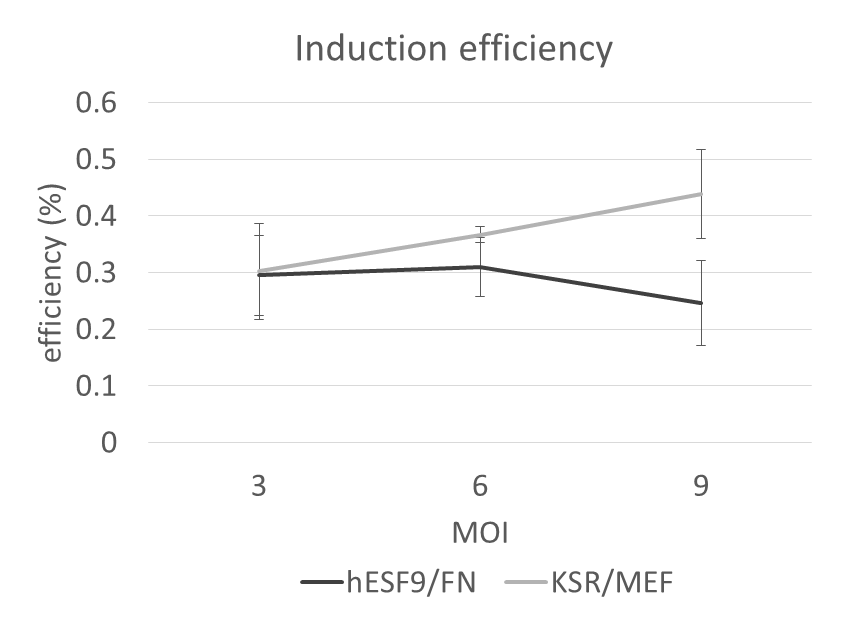


　　Figure S2: hiPSC generation from DPCs in serum- and feeder-free culture conditions.

1. Transduced DPCs (DPC-M) were cultured on fibronectin (FN) with hESF9 medium or on MEF with KSR-based conditions at MOI=3, 6, 9. After 20 days, iPS colony were picked up and sub-cultured on fibronectin.
2. Numbers of ALP-positive colonies in relation to multiplicity of infection (MOI=3, 6, 9) at a density of 1.0x10^4^ per 6-well plate on fibronectin. The number of ALP-positive hESC-like colonies was counted. The efficiency of iPSC colony generation was dependent upon the dosage of virus MOI used for gene introduction in serum-supplemented condition (KSR/MEF). Whereas, the efficiency was not dose-dependent manner in serum- and feeder-free culture conditions (hESF9/FN). (n=10)

**Supplementary Figure-3**

**hiPSCs derived from DPCs in completely serum- and feeder-free culture conditions.**

**
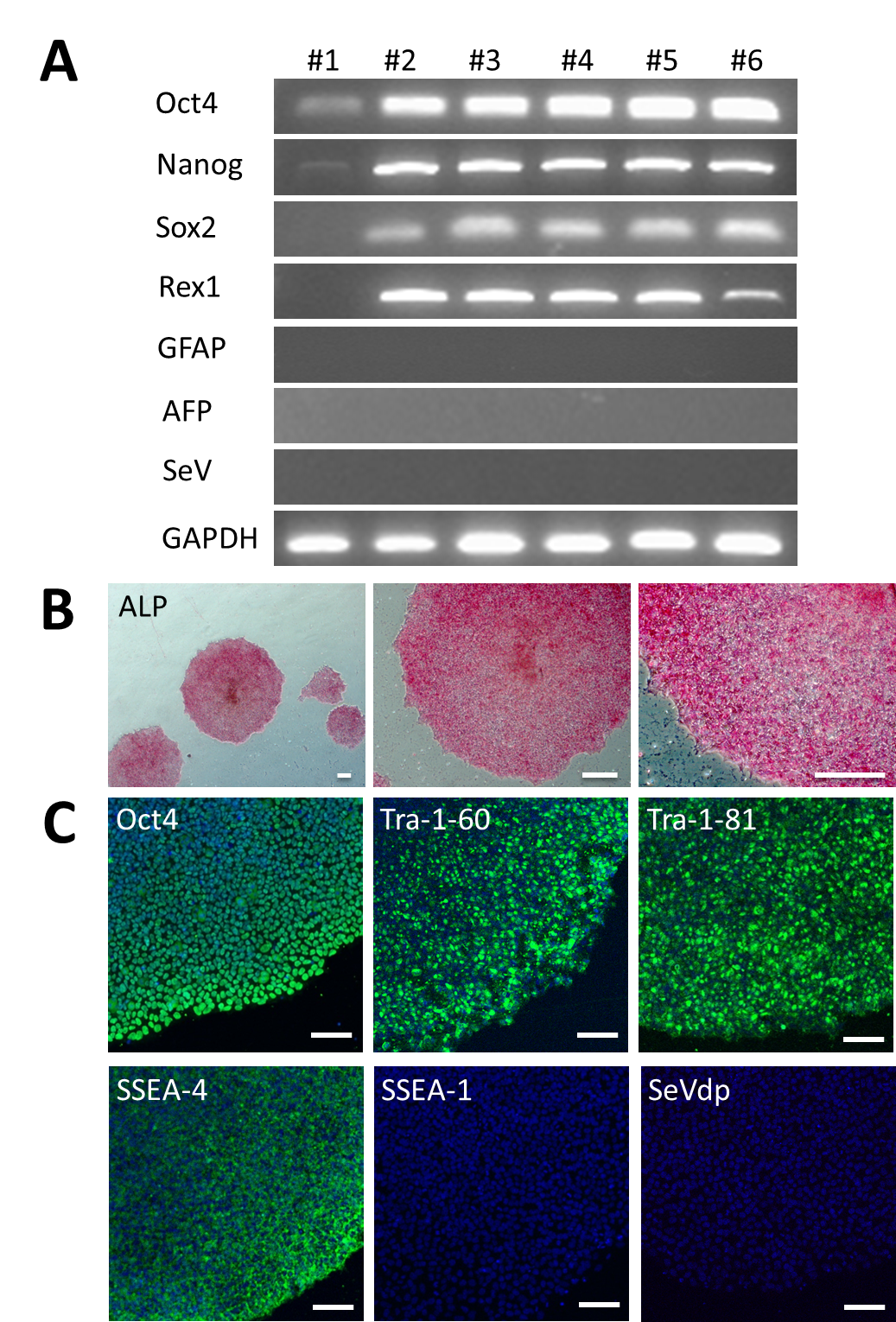
**

FigureS3: hiPSCs derived from DPCs in completely serum- and feeder-free culture conditions.

1. Expression of ES cell marker genes in iPSCs derived from DPCs.

#1: Dental Pulp cell (DPC-H): passage 1 = before infection

#2: SeV-H-iPS (clone25): passage 38 = serum-free condition

#3: SeV-H-iPS (clone25): passage 61 = serum-free condition

#4: SeV-H-iPS (clone35): passage 38 = serum-free condition

#5: SeV-H-iPS (clone35): passage 71 = serum-free condition

#6: SeV-H-iPS (clone23): passage 7 = serum-supplemented condition

DPC-H-derived iPS cells were designated SeV-H-iPS

1. ALP activity of generated iPSCs

The ALP activity was detected (SeV-H-iPS clone25 passage 33).

Bars indicate 200 μm.

1. Immunocytochemistry of pluripotency marker proteins

SeV-H-iPS clone25 grown under hESF9-based culture conditions for 33 passages were fixed and reacted with antibodies (Oct4, Tra-1-60 and Tra-1-81, SSEA-4, SSEA-1, SeVdp). Binding of these antibodies was visualized with Alexa Fluor® 488-conjugated secondary antibodies (green). Nuclei were stained with DAPI (blue).

Scale bars represent 100μm.

**Supplementary Figure-4**

**Characterization of SeV-iPSCs generated in serum-, feeder- and integration-free defined culture conditions.**

**
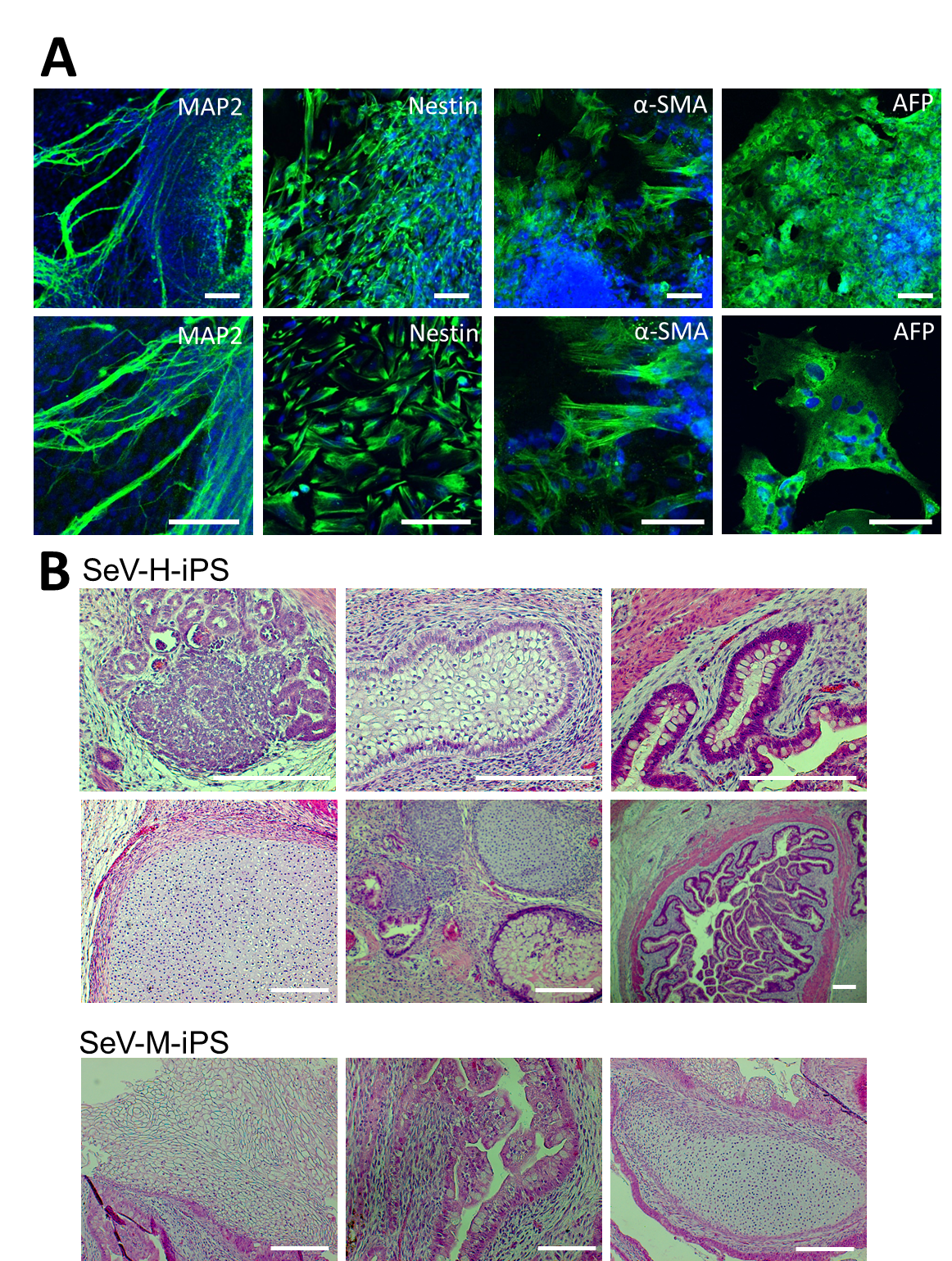
**

Figure S4: Characterization of SeV-iPSCs generated in serum-, feeder- and

integration-free defined culture conditions.

1. Differentiation was performed using embryoid body formation, and the differentiated

iPSCs (SeV-H-iPS clone25) were fixed and reacted with antibodies.

Shown were immunocytochemistry of MAP-2, Nestin, α-smooth muscle actin (α-SMA), and α-fetoprotein (AFP). Binding of these antibodies was visualized with Alexa Fluor® 488-conjugated secondary antibodies (green). Nucleuses were stained with DAPI. (passage 50)

Bar indicates 100μm.

B) Teratoma formation of hiPSCs in the defined culture conditions in SCID mice.

Teratomas were generated in SCID mice (CB17/Icr-Prkdcscid/CrlCrlj) from SeV-H-iPS and SeV-M-iPS. Histological analysis with H-E staining demonstrated that teratomas formed from iPSCs cultured in hESF9-based conditions contained derivatives of all three germ layers.

Scale bars represent 200 μm.

**Supplementary Figure 5**

**PCR analysis of genomic DNA confirms integrations of the transgenes.**

**
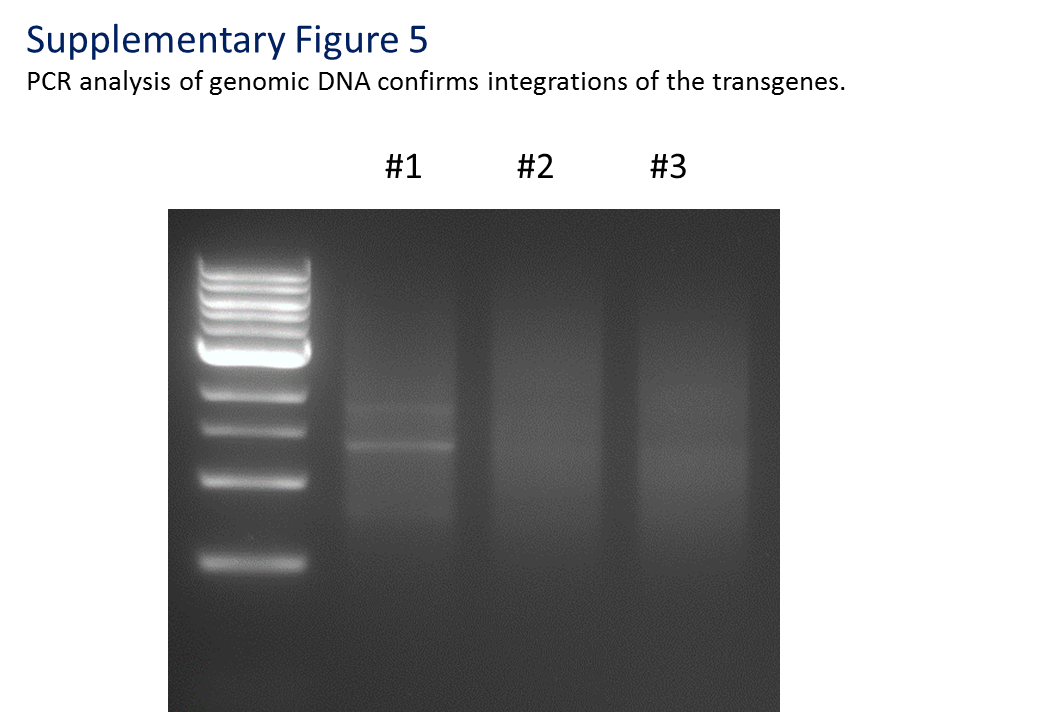
**

Figure S5: PCR analysis of genomic DNA confirms integrations of the transgenes in retrovirus-induced DPC-H-iPSCs, but not in SeV-H-iPSCs.

Genomic DNA was isolated from SeV-H-iPSCs and DPC-H-iPSCs.

SeV-H-iPSCs were generated from DPC-H using SeVdp (KOSM) vector, whereas DPC-H-iPSCs were generated from DPC-H using retrovirus (Oct4, Sox2, Klf-4, c-Myc). These iPSCs were derived from DPCs of same person. Reactions were performed with pMXs primers described in Supplementary Table S2.

#1 DP-H-iPSC clone30 passage 50

#2 SeV-H-iPSC clone25 passage 40

#3 DPC-H passage 1

**Supplementary Figure 6**

**Karyotype analysis of Q-banding.**


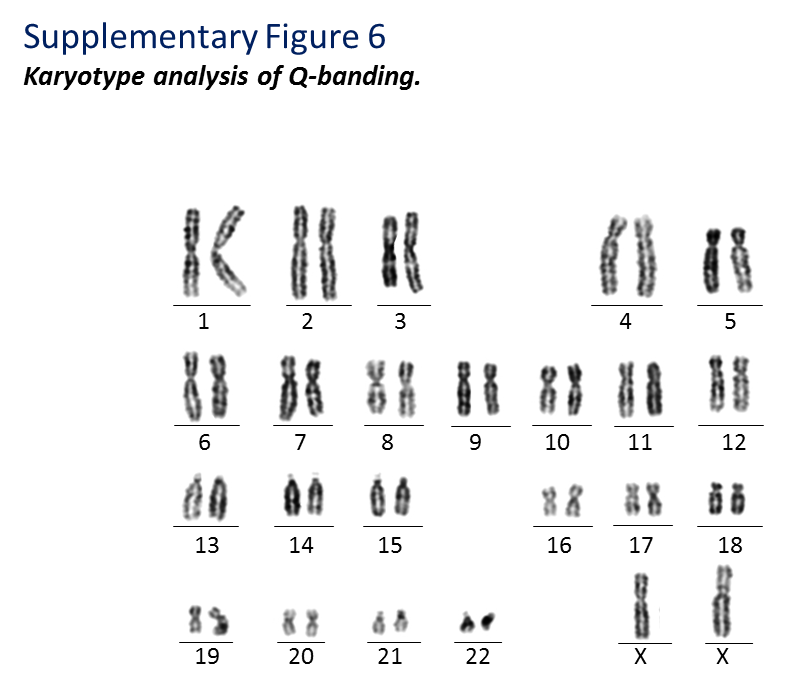


Figure S6: Karyotype analysis of Q-banding.

SeV-H-iPS clone 35 at passage 92 generated in hESF9 had a normal diploid 46, XX karyotype.

**Supplementary Figure 7**

**Characterization of CCD-iPSCs generated in serum-, feeder-, and integration-free culture conditions.**

**
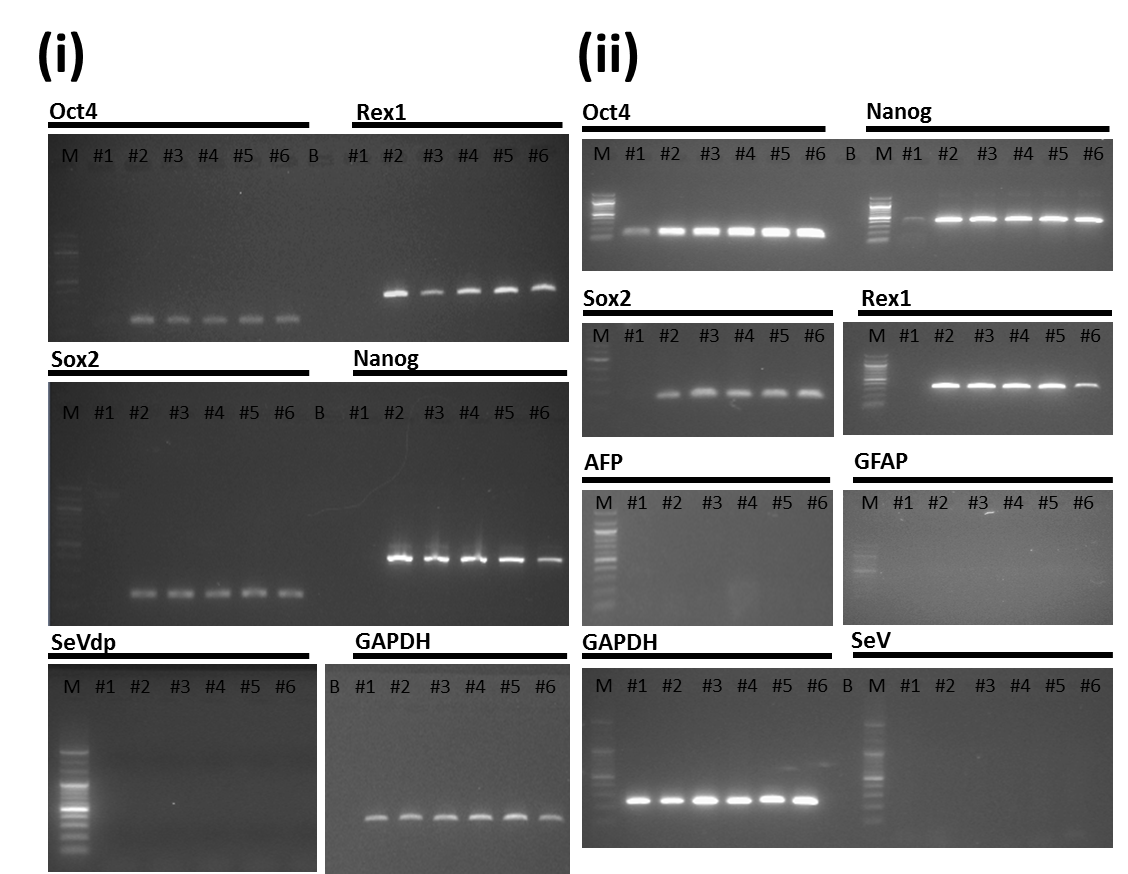
**

Figure S7: Characterization of CCD-iPSCs generated in serum-, feeder-, and integration-free culture conditions.

Full-length images of gels presented in Figures 4 (i) and Figure S3 (ii).

Expression of ES cell marker genes in iPSCs derived from CCD-DPCs.

M :marker

B :Blank

**Supplementary Table S1: STR analyses of DPC-derived iPSCs.**

| allele data | | |  |  |  |  |  |  |
| --- | --- | --- | --- | --- | --- | --- | --- | --- |
| Locus | DPC-H | | SeV-H-iPS clone25passage40 | | DPC-M | | SeV-M-iPS clone6 assage6 | |
| **D3S1358** | **15** | **17** | **15** | **17** | **15** | **18** | **15** | **18** |
| **TH01** | **7** | **9** | **7** | **9** | **6** | **7** | **6** | **7** |
| **D21S11** | **31** | **31.2** | **31** | **31.2** | **29** | **30** | **29** | **30** |
| **D18S51** | **14** | **20** | **14** | **20** | **16** | **18** | **16** | **18** |
| **Penta_E** | **11** | **17** | **11** | **17** | **11** | **12** | **11** | **12** |
| **D5S818** | **11** |  | **11** |  | **11** |  | **11** |  |
| **D13S317** | **9** |  | **9** |  | **11** | **13** | **11** | **13** |
| **D7S820** | **11** | **12** | **11** | **12** | **10** | **13** | **10** | **13** |
| **D16S539** | **13** |  | **13** |  | **10** | **11** | **10** | **11** |
| **CSF1PO** | **9** | **12** | **9** | **12** | **10** | **11** | **10** | **11** |
| **Penta_D** | **9** | **13** | **9** | **13** | **10** | **13** | **10** | **13** |
| **AMEL** | **X** |  | **X** |  | **X** | **Y** | **X** | **Y** |
| **vWA** | **18** | **20** | **18** | **20** | **16** | **18** | **16** | **18** |
| **D8S1179** | **12** | **13** | **12** | **13** | **12** | **14** | **12** | **14** |
| **TPOX** | **8** | **11** | **8** | **11** | **8** |  | **8** |  |
| **FGA** | **21** | **24** | **21** | **24** | **23** | **24** | **23** | **24** |
|  |  |  |  |  |  | | | |
| Locus | CCD-DPC | | CCD-SeV-iPS  clone1 passage3 | |  | | | |
| **D3S1358** | **16** | **17** | **16** | **17** |  | | | |
| **TH01** | **7** | **9** | **7** | **9** |  | | | |
| **D21S11** | **30** |  | **30** |  |  | | | |
| **D18S51** | **14** |  | **14** |  |  | | | |
| **Penta_E** | **10** | **12** | **10** | **12** |  | | | |
| **D5S818** | **9** | **11** | **9** | **11** |  | | | |
| **D13S317** | **10** | **12** | **10** | **12** |  | | | |
| **D7S820** | **10** | **11** | **10** | **11** |  | | | |
| **D16S539** | **9** | **11** | **9** | **11** |  | | | |
| **CSF1PO** | **12** | **14** | **12** | **14** |  | | | |
| **Penta_D** | **9** | **13** | **9** | **13** |  | | | |
| **AMEL** | **X** |  | **X** |  |  | | | |
| **vWA** | **14** | **17** | **14** | **17** |  | | | |
| **D8S1179** | **13** | **14** | **13** | **14** |  | | | |
| **TPOX** | **8** | **11** | **8** | **11** |  | | | |
| **FGA** | **21** | **24** | **21** | **24** |  | | | |

**Supplementary Table S2: Primers used in this study.**

| Gene Name | Primer sequence |
| --- | --- |
| endogenous Sox2 | 5'- GGG AAA TGG GAG GGG TGC AAA AGA GG-3' |
|  | 5'- TTG CGT GAG TGT GGA TGG GAT TGG TG -3' |
| endogenous Nanog | 5'- CAG CCC CGA TTC TTC CAC CAG TCC C -3' |
|  | 5'- CGG AAG ATT CCC AGT CGG GTT CAC C -3' |
| endogenous Oct3/4 | 5'- GAC AGG GGG AGG GGA GGA GCT AGG -3' |
|  | 5'- CTT CCC TCC AAC CAG TTG CCC CAA AC -3' |
| Rex-1 | 5'- CAG ATC CTA AAC AGC TCG CAG AAT -3' |
|  | 5'- GCG TAC GCA AAT TAA AGT CCA GA -3' |
| Esg-1 | 5'- ATA TCC CGC CGT GGG TGA AAG TTC -3' |
|  | 5'- ACT CAG CCA TGG ACT GGA GCA TCC -3' |
| AFP | 5'- GAA TGC TGC AAA CTG ACC ACG CTG GAA C -3' |
|  | 5'- TGG CAT TCA AGA GGG TTT TCA GTC TGG A -3' |
| GFAP | 5'- GGC CCG CCA CTT GCA GGA GTA CCA GG -3' |
|  | 5'- CTT CTG CTC GGG CCC CTC ATG AGA CG -3' |
| SeVdp NP | 5’- AGA CCC TAA GAG GAC GAA GA -3’ |
|  | 5’- ACT CCC ATG GCG TAA CTC CAT AGT G -3’ |
| GAPDH | 5'- TGA TGA CAT CAA GAA GGT GGT GAA G -3' |
|  | 5'- TCC TTG GAG GCC ATG TGG GCC AT -3' |
| pMX-S1811 | 5'- GAC GGC ATC GCA GCT TGG ATA CAC -3' |
| pMXs-AS3200 | 5'- TTA TCG TCG ACC ACT GTG CTG CTG -3' |
| GAPDH ; glyceraldehyde-3- phosphate dehydrogenase | |
